# Latent Diversity in Human Concepts

**DOI:** 10.1162/opmi_a_00072

**Published:** 2023-03-09

**Authors:** Louis Marti, Shengyi Wu, Steven T. Piantadosi, Celeste Kidd

**Affiliations:** University of California, Berkeley, Berkeley, CA

**Keywords:** concepts, metacognition, individual differences, ordinary meaning

## Abstract

Many social and legal conflicts hinge on semantic disagreements. Understanding the origins and implications of these disagreements necessitates novel methods for identifying and quantifying variation in semantic cognition between individuals. We collected conceptual similarity ratings and feature judgements from a variety of words in two domains. We analyzed this data using a non-parametric clustering scheme, as well as an ecological statistical estimator, in order to infer the number of different variants of common concepts that exist in the population. Our results show at least ten to thirty quantifiably different variants of word meanings exist for even common nouns. Further, people are unaware of this variation, and exhibit a strong bias to erroneously believe that other people share their semantics. This highlights conceptual factors that likely interfere with productive political and social discourse.

## INTRODUCTION

Even when two individuals use the same word, they do not necessarily agree on its meaning. Disagreements about meaning are common in debates about terms like “species” (Zachos, 2016), “genes” (Stotz et al., 2004), or “life” (Trifonov, 2011) in biology; “curiosity” (Grossnickle, 2016), “knowledge” (Lehrer, 2018), or “intelligence” (Sternberg, 2005) in psychology; and “measurement” in physics (Wigner, 1995). Ernst Mach and Albert Einstein even disagreed about what constitutes a “fact” (de Waal & ten Hagen, 2020). In contemporary society, social issues often hinge on the precise meaning of terms like “equity” (Benjamin, 2019), “pornography” (Stewart, 1964), “peace” (Leshem & Halperin, 2020), or the “right to bear arms” (Winkler, 2011). Sometimes these debates are settled by fiat—for example, the U.S. Supreme court decided that a tomato counted as a vegetable (not a fruit) for tax purposes because the law should follow the “ordinary meaning” of words rather than their botanical meaning (see Goldfarb, 2021; Nix v. Hedden, 149 U.S. 304, 1893).

Despite the frequency of such terminological debates, these conflicts have not been characterized using cognitive psychology methods. Multidimensional scaling methods (Shepard, 1962a, 1980; Torgerson, 1952) have been used in psychometrics to study individual differences in concepts and their relational or geometric structure (Bocci & Vichi, 2011; Carroll & Chang, 1970; McGee, 1968; Takane et al., 1977; Tucker & Messick, 1963). For example, Tucker and Messick (1963) used a multidimensional scaling analysis to infer consistent individual differences in perceptions and judgements of distance estimates. This approach avoided the pitfalls of more common methods of using group averages in judgements to draw general conclusions about a theoretical “average person”—which the authors rightly observe may not actually resemble any actual participant at all. Instead the authors’ multidimensional scaling analysis demonstrated distinct and consistent viewpoints across individuals. Recent implementations capitalize on the advantages of generative Bayesian statistical inference in order to characterize individual differences and the importance of specific dimensions (Gelman et al., 2013; Kruschke, 2010; Okada & Lee, 2016). Prior work has also demonstrated the existence of individual differences in conceptual judgements (Barsalou, 1987; Hampton & Passanisi, 2016; Koriat & Sorka, 2015), but not quantified the degree of variation for concepts across the population. Verheyen and Storms (2013) found subgroups of categorizers who emphasize different attributes (e.g., vagueness or ambiguity) when determining membership. Bush characterized multiple dimensions of feeling adjectives and found individual differences in the perception of feelings (Bush, 1973). Labov (1973) observed that conceptual category boundaries for cups and bowls could vary across individuals in the two dimensional space of height and width. Labov found greater disagreement in atypical cases compared to typical exemplars, a finding which holds in other conceptual domains (McCloskey & Glucksberg, 1978). Differences in training can result in conceptual variation—for instance, philosophers view “knowledge” differently than others (Starmans & Friedman, 2020).

These data suggest that conceptual variability relates to real world experiences, but does not tell us how commonly conceptual disagreements occur in semantic cognition. If conceptual variability is commonplace, that would suggests the variability is fundamental feature of our conceptual systems, perhaps an inevitable byproduct of the substantial experiential differences people accumulate throughout their lives. Indeed, two people may experience the same event but process it differently due to individual differences in cognition or prior experience, influencing concept formation. Such a finding would also implicate conceptual misalignment as an underappreciated explanation for a broad range of disagreements in theoretical and applied fields.

One challenge for understanding variation in concepts is that there are no complete accounts of human conceptual representation (see, e.g., Laurence & Margolis, 1999; Murphy, 2004) and therefore people’s representations must be measured indirectly. Here, we ask participants about the conceptual representations they attach to words, building on the productive history of studying concepts via linguistic labels (Lupyan & Thompson-Schill, 2012; Rosch & Lloyd, 1978). As a quantitative measure, we collected conceptual ratings (Barsalou, 1989; Landauer & Dumais, 1997; Mikolov et al., 2013; Shepard, 1962a, 1962b, 1980) of both *similarity* judgements and *features*. The similarity task asked people to judge whether, for example, a penguin is more similar to a chicken or a whale. The feature experiment first freely elicited features from one set of participants, and then asked a group of participants to rate the applicability of each of the elicited features to each concept. For example, participants judged whether a penguin was “majestic”. We note that similarity judgements and features have well-known limitations, including for example that similarities are sensitive to the respects with which similarity is computed (Gentner & Markman, 1997; Markman & Gentner, 1993; Medin et al., 1993; Tversky & Gati, 1978); however, for our purposes of studying variability, it is less important that features and similarities do not completely characterize people’s conceptual knowledge. Differences in features and similarities still indicate that there are *some* underlying differences.

We gathered these ratings in two domains: common animals and politicians. The animal domain allows us to characterize diversity for high-frequency nouns which may be most likely to be shared. We contrast this with politicians, which might vary among individuals with distinct political beliefs. Prior work for example has found that concepts and language concerning morality differ with political view (Frimer, 2020; Graham et al., 2009). The experiment also asked participants to make the same similarity ratings and feature judgements multiple times. This allowed us to determine reliability of ratings. We used this to test the possibility that people shared the same concepts, but that the concepts were just noisily measured. Our main results showing multiple concepts in the population therefore reflect statistical evidence of multiple concepts above and beyond response inconsistencies.

**Figure 1.**
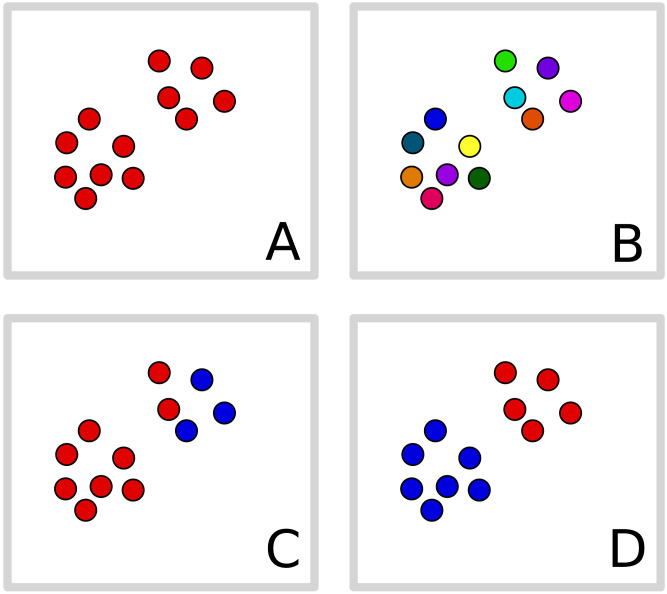
**Hypothetical clustering of response vectors, here visualized in 2D.** The simplest solution is to put all points into the same cluster (A), but then responses (locations) are not well-explained by clusters. If each point is in a separate cluster (B) then each point is perfectly predicted by the cluster, but the solution is complex. A compromise like (D) finds a small number of clusters that adequately explain the data. The correct clustering (D) will be preferred over alternatives even with the same number of total clusters which fit the data less well (C).

Our primary analysis uses a non-parametric Bayesian clustering model in order to infer *how many* types of each concept (clusters) were likely to be present in our sample. For example, how many different concepts of “finch” did people exhibit based on their similarity judgements? This clustering method does not presuppose a fixed number of clusters, but infers a distribution of what clusters are likely present based on the data by balancing two competing pressures. First, the model is biased to prefer a small number of clusters since this is a *simpler* theory. In the absence of data, the number of clusters should not be “multiplied without necessity” (i.e., Ockham’s Razor). Second, the model prefers clusterings that *explain* the data. Here, that means that the inferred clustering should predict responses in the sense that two individuals in the same cluster should give similar responses. This is illustrated in Figure 1, where we can abstractly imagine possible clusterings (colors) of responses, which are here abstractly visualized in two dimensions. Clusterings like (A) are too simple; (B) is too complex; (C) is simple but does not do a good job of explaining the data; intuitively, (D) is a good solution. In essence, the model stochastically infers the colors in this figure from the responses, providing us with a statistical estimate of the number of clusters. Specifically, we use a non-parametric scheme (Anderson, 1991; Gershman & Blei, 2012; Pitman, 1995) which translates both the pressures for simplicity and fit into probability theory, and then balances—optimally, in a precise sense–between the two (see Materials and Methods). This inference critically depends on the reliability of subject responses and only using this model are we able to infer the number of clusters that likely generated the data.

We are also interested in the number of clusters present in the population beyond our experimental sample. To quantify this, we used an estimator from population ecology (Chao & Chiu, 2016). This model is used in species count estimation (Bunge & Fitzpatrick, 1993), where one might sample animals, observe how many of each species were collected in the sample, and estimate the total number of species present in the world (see also Gale & Sampson, 1995; Good, 1953). We use the most-likely (maximum a posteriori) clustering of individuals in the Bayesian model in order to estimate the total number of concepts present in the world, beyond of our sample.

Finally, we asked participants to report what proportion of other people they expected to agree with them about their similarity judgements. We compared these reports to the observed agreement levels.

These tools allow us to test a variety of novel hypotheses about variation in human conceptual systems. First, by examining the estimated number of clusters (both in the sample and the general population), we evaluate how many measurably distinct representations can be found in the population. This estimate is conservative since it is derived by similarities to a relatively small number of other nouns; larger and more detailed experiments might reveal more conceptual variation. Despite this conservativity, our results reveal substantial variation, with more variation in politicians than animals. Moreover, because our inference relies on a probabilistic model which incorporates multiple-measurement reliability, these clusters cannot be due to measurement noise. Finally, the results show that people are generally unaware of these differences: people expect that others will answer the same way that they do more often than is true. This lack of awareness suggests that latent variation may underlie disagreement on broader social and political issues.

## MATERIALS AND METHODS

Experiment 1 was run using a custom built web interface on Amazon Mechanical Turk on 8/20/19 through 8/22/19 (animals) and 9/11/19 through 9/12/19 (politicians). Participants were instructed to “decide which [animal/politician] is more similar to [target concept]” and “asked to guess how many people out of 100 would agree with you.” All participants were required to be from the U.S. and have a minimum 95% approval rating from previous tasks. Experiment 2 was run on 04/23/21 through 05/09/21 (animals) and 05/13/21 through 05/17/21 (politicians) using Prolific and Qualtrics. Participants were all above 18 years old, fluent English speakers, and physically present in the United States based on pre-screening questions. Responses were recorded on a secure server and no participants were excluded from data analysis. All participants were paid at a rate of $10 an hour. This study was approved by the Committee for Protection of Human Subjects at University of California, Berkeley. Informed consent was obtained from all subjects. All methods were performed in accordance with relevant guidelines and regulations at University of California, Berkeley (CPHS # 2018-12-11675).

### Clustering Methods

Responses were clustered using a non-parametric, Bayesian clustering model, a “Chinese restaurant process.” If *x* = 〈*x*_1_, *x*_2_, …, *x*_*k*_〉 denotes the number of subjects in each cluster (for a given word), and *n* denotes the total number of subjects, this model assigns *x*, a partition on individuals, a prior of

$$\frac{\Gamma \left(\theta \right)\cdot \theta^{k}}{\Gamma \left(n+\theta \right)}\cdot \prod_{i=1}^{k}\Gamma \left(x_{i}\right),$$ (1)

where we use *θ* = 1, to characterize how strongly the model prefers fewer clusters. Within each cluster, we use a Beta-Binomial likelihood where subjects assigned the same cluster are assumed to generate the same latent vector of answer probabilities, with each cluster’s probability vector marginalized out. Thus, if *a*_*ij*_ and *b*_*ij*_ are the number of each type of response to question *j* in cluster *i*, and *q* the number of questions, then the marginal likelihood of the responses is,

$$\prod_{i=1}^{k}\prod_{j=1}^{q}\frac{B\left(a_{ij}+\alpha ,b_{ij}+\alpha \right)}{B\left(\alpha ,\alpha \right)},$$ (2)

where *B*(*a*, *b*) = Γ(*a*) · Γ(*b*) / Γ(*a* + *b*). Here, *α* characterizes the noise level assumed by the likelihood. We set the single likelihood parameter *α* = 0.16 such that two samples from a Bernoulli with parameter *p* ∼ *Beta*(*α*, *α*) agreed with each other with probability 0.88, which is the proportion of time subjects’ second and first responses agreed (analysis of the dependence of the results to the assumed *α* is in Supplementary Materials).

Inference was run using a Gibbs sampler, using both the prior (Eq. 1) above and a uniform prior over clusters. All runs used the same likelihood (Eq. 2). The sampler was initialized with a configuration where each individual started in the same cluster. This sampling method requires iterations of burn-in before it converges to a stable set posterior distribution. We assessed convergence using multiple runs and ensured that chains arrived at the same solution. Figure 7 in Supplementary Materials shows the convergence of three chains for each concept over 500 iterations (one iteration is a Gibbs sweep through the whole population). We discarded the first 100 samples from each run as burn-in.

### Ecological Estimator

Finally, we use an ecological estimator from Chao and Chiu (2016), extending a previous estimator (Colwell & Coddington, 1994), in order to approximate the total number of concepts in the population. This estimator uses the total number of observed clusters (concepts) and the total number of sampled individuals in order to estimate how many concepts were likely unobserved. The method is a relative of Good-Turing estimation (Good, 1953), and also depends on the number of clusters containing a single person, among additional terms. For this we use our maximum a posteriori Bayesian clustering. Let *f*_*i*_ denote the number of clusters containing *i* individuals, then the estimator $\hat{S}$_*Chao*1_ is given by,

$${\hat{S}}_{\text{Chao}1}=\left\{\begin{array}{ll} S_{obs}+\frac{\left(n-1\right)}{n}\frac{f_{1}^{2}}{2f_{2}}, & \text{if}\;f_{2}>0\\ S_{obs}+\frac{\left(n-1\right)}{n}\frac{f_{1}\left(f_{1}-1\right)}{2}, & \text{if}\;f_{2}=0. \end{array}\right.$$ (3)

Here, *S*_*obs*_ denotes the number of observed clusters and *n* is the number of participants sampled. The estimator we used (Chao & Chiu, 2016) adjusts $\hat{S}$_*Chao*1_ to yield $\hat{S}$_*iChao*1_,

$${\hat{S}}_{\text{iChao}1}={\hat{S}}_{\text{Chao}1}+\frac{\left(n-3\right)}{n}\cdot \frac{f_{3}}{4f_{4}}\cdot \max\left(f_{1}-\frac{\left(n-3\right)}{\left(n-1\right)}\frac{f_{2}f_{3}}{2f_{4}},0\right).$$ (4)

## EXPERIMENT 1

We recruited 1,799 participants on Amazon Mechanical Turk. Half were asked to make similarity judgements about animals (finch, robin, chicken, eagle, ostrich, penguin, salmon, seal, dolphin, whale) and the other half to make judgements about U.S. politicians (Abraham Lincoln, Barack Obama, Bernie Sanders, Donald Trump, Elizabeth Warren, George W. Bush, Hillary Clinton, Joe Biden, Richard Nixon, Ronald Reagan). Each participant was randomly assigned to a single target from one domain (e.g., “finch”), presented with 36 unique pairs of other objects in the domain (drawing from the 10 objects in each domain), and asked which was more similar to the target. Thus, participants responded to queries such as “Which is more similar to a finch, a whale or a penguin?” Each trial was shown twice (for a total of 72 trials) in order to measure response reliability (calculated as the percentage of trial-pairs with identical responses) and detect trial-by-trial idiosyncratic features of stimuli. To quantify metacognitive awareness of diversity, participants were also simultaneously asked on every trial to guess how many people out of 100 would agree with their response.

We coded each participant’s responses to a single word as a binary vector, corresponding to theforced-choice similarity rating between every other pair of items. In modeling, we assumed that individual vectors were sampled from a collection of latent clusters that specified an average response vector. We used a nonparametric Bayesian technique called a Chinese Restaurant Process (Anderson, 1991; Gershman & Blei, 2012; Pitman, 1995), to model a posterior distribution on the number of clusters for each word independently, assuming a reliability given by the overall average reliability. We note this clustering model works in the space of response vectors, not in the lower-dimensional psychological space itself; thus, our approach does not explicitly model correlations that may exist between items, but also does not require us to make assumptions about the dimensionality or metric properties of the latent space. This technique permits us to find a distribution over the number of clusters present in the population, taking into account both the reliability of individual responses and uncertainty about the latent response vector characterizing each cluster (e.g., *what* each participant’s “finch” cluster corresponds to in terms of similarities). The model builds in a prior preference for fewer clusters but we also present results with no such prior. The maximum a posteriori clusterings found in sampling were additionally put through a species-count estimator which estimates the true number of clusters present in the global population, beyond our finite sample size (Chao & Chiu, 2016). This estimator uses sampled individuals which are observed to fall into a distribution of species and estimates the total number of species (here, clusters) in the population at large.

### Experiment 1 Results

The overall subject reliability was 88% (see Figure 6 in Supplemental Materials), indicating subjects are both not responding with random guesses, nor are they responding with ad hoc responses that vary from trial to trial. Subject responses likely reflect stable aspects of how they conceptualize these concepts throughout the context of the experiment. On the other hand, the average *inter*subject reliability across all concepts was 50% (ranging from 33% to 62% with no significant differences between animals or politicians), meaning two people picked at random are just as likely to disagree as agree for a typical concept judgement. Intersubject reliability was 50% for both the first and second judgements. We kept the first judgement for analysis.

**Figure 2.**
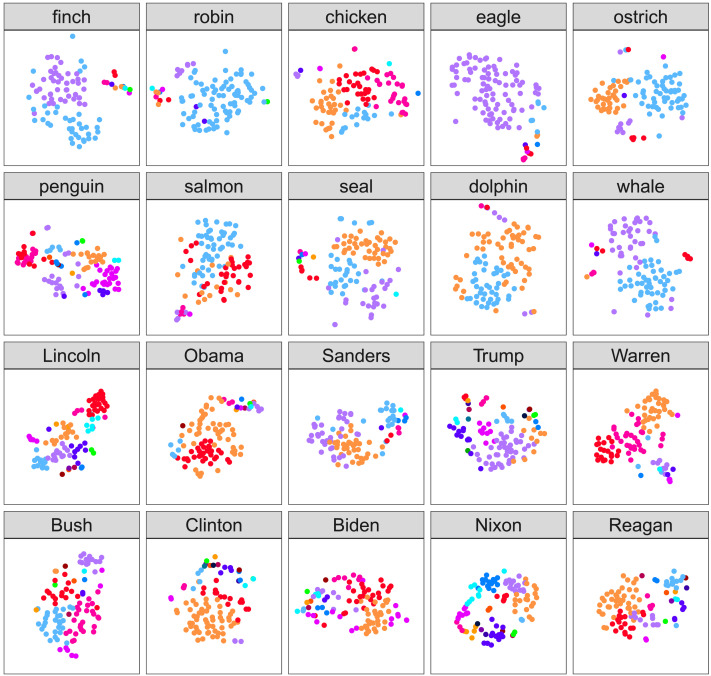
**Distances between participants’ conceptual representations of each target concept using distributed stochastic neighbor embedding.** In this visualization, the distances between two points approximate the distance between their full rating vectors. Each plot is on the same scale. Additionally, each data-point is colored with the cluster they were assigned to in our clustering analysis, showing that the t-SNE clustering finds similar groupings.

Figure 2 shows a visualization of participants’ similarity judgements using distributed stochastic neighbor embedding (t-SNE) (van der Maaten & Hinton, 2008). This technique places individual participants’ response vectors in a 2D plane such that nearby participants give similar response vectors. The closer two points are together, the more closely their concepts align; however, these scales are relative and cannot easily be compared across plots. Points in this plot have been colored according to the maximum a posteriori assignment of participants to clusters according to the clustering model, which was run independently from t-SNE, and thus convergence serves as a check on both methods. This figure illustrates that two independent methods provide convergent characterizations of how people are distributed in the space since each color (generated according to the clustering model) tends to be in a single spatial position (generated by t-SNE). Note that the color assignments do not perfectly match spatial arrangements, likely due to t-SNE dimensionality reduction and different trade-offs being applied to edge-case participants by our algorithm and t-SNE.

**Figure 3.**
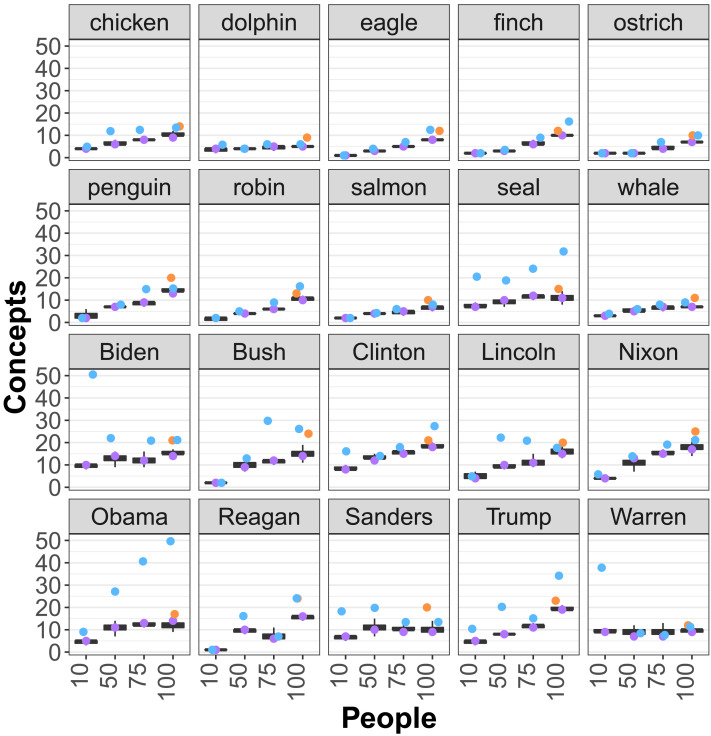
**Estimated number of concepts (*y* axis) depending on the number of people sampled (*x* axis).** Boxes show the median 50% quantiles of the number of unique concepts. Purple data points are the number of clusters for the *maximum a posteriori* clustering. Orange data points are the number of clusters for the MAP clustering with a uniform prior. Blue data points show the ecological estimator using the MAP clustering.

To understand the number of concepts in the population, we first look at the posterior distribution over the *number* of clusters inferred. Figure 3 shows the estimated number of conceptual kinds (*y* axis) for each semantic domain (subplot), as a function of the number of participants included (*x* axis). This figure shows that as our sample size increases from 10 to 100 individuals per concept, the number of estimated concepts reaches 9 to 19 for politicians and 5 to 13 for animals. The maximum a posteriori clustering (in purple) and the ecological estimator (in blue) are in the range of 5–50 latent concepts in the population, and are higher for politicians than for animals. We find similar ranges even if we use a prior which is uniform on the clusterings (orange).

We note that the number of inferred concepts is not necessarily monotonically increasing in the number subjects, since additional subjects may shape the geometry of the space (e.g., providing evidence that two separate clusters are actually one wider cluster). In addition, most of the latent diversity can be found in small numbers of subjects—even distinct clusters can be found when examining 50 individuals. The point at which each subplot levels off is due to a combination of the reliability of individual responses, the number of items we sampled (sampling less results in fewer concepts), and the true number of concepts in the population. However, limited reliability and a finite number of items mean that our analysis is likely to *under*-estimate the number of clusters.

**Figure 4.**
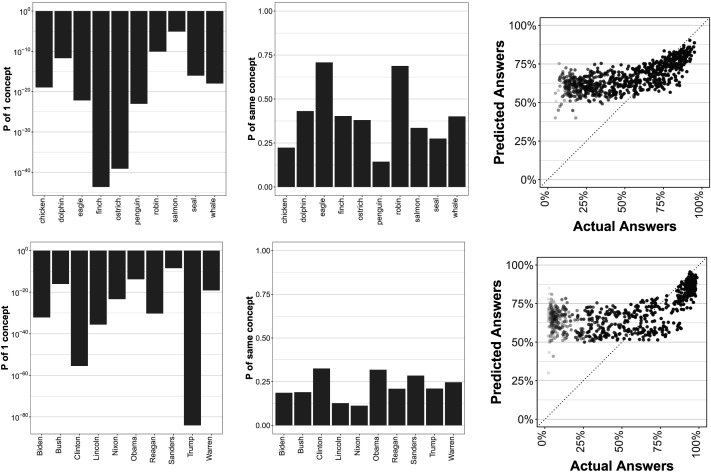
**Left: Probabilities that only *a single conceptual representation* for each word exists, with log-axis, showing near zero probability for all words, especially politicians.** Middle: Probability that two random individuals will share the same table (i.e., concept), showing low rates of agreement for politicians and slightly higher for animals. Right: Predicted answers (*y* axis) vs. actual answers (*x* axis), showing people tend to over-estimate others’ rate of agreement compared to the truth (line *y* = *x*).

Figure 4 shows the probability that the population contains only *one* concept for each word, according to the clustering model. Because samples rarely contained a single cluster, we used a normal approximation to compute this probability, using the mean and standard deviation of number of samples according to the posterior distribution on clustering. Political words are far less likely to have a single meaning than animal words, matching the patterns in the number of clusters in Figure 3. Generally, this provides strong statistical support to the idea that there are multiple meanings in the population for these terms, despite the fact that these multiple concepts all have the same word. However, if the distribution of participants to meanings tends to be heavily skewed (e.g., most participants have the same meaning), then this diversity might be relatively inconsequential. Figure 4 shows the probability that two randomly chosen individuals will have the same concept in this analysis, which is a relatively robust statistic since it depends largely on the frequency of the most common concepts for each word rather than the tails of the distribution. This agreement probability averages to around 14–70% for animals and 13–33% for politicians. This indicates that *most* individuals one encounters will tend to have a measurably different conceptual representation. Again, this is likely to over-estimate true rate of agreement since we only tested a small number of questions.

Most importantly, our results show that *people are generally not aware of these differences*. Figure 4 shows the agreement rate on responses (*x* axis) compared to people’s predicted estimates of agreement (*y* axis). If people understood the population’s variation in responses, the trials shown in this plot would all fall along the *y* = *x* line. Instead, this figure shows that for most of the range of actual agreement (e.g., ∼0%–80%) people tend to consistently believe that about 2/3 of participants will agree with them, no matter what true proportion actually do. This is true even for the lowest agreement responses: most participants believe their response is in the majority even when essentially 0% of other participants agree with them. This is unlikely to be due to a failure to engage this aspect of the task because participants *do* reliably increase their estimates on the highest agreement items (e.g., ∼80%–100%), which results in a reliable rank-order correlation overall (Spearman’s *ρ* = 0.45, *p* < 0.001). The increase, though, is not well-calibrated to the population variation. Moreover, these patterns likely reflect meta-cognitive limitations (Goldman, 2006; Gopnik & Meltzoff, 1997; Wimmer & Perner, 1983) rather than differences in effort or motivation because these trials were interspersed with the main task, which had very high within-subject reliability.

## EXPERIMENT 2

Experiment 2 consisted of two parts: feature elicitation and feature rating. In feature elicitation, we recruited 16 registered users on Prolific. Half of the participants were asked to list 10 single-word adjective features for each of the 10 animals in Experiment 1. The other half were asked to list 10 single-word adjective features for each of the 10 U.S. politicians in Experiment 1. We kept all features that were mentioned more than once after removing non-adjectives, inappropriate words, and typos, as well as combining synonyms.

Then, 1,000 registered users on Prolific were asked to rate either 105 animal features or 105 politician features from the feature elicitation experiments. Each participant was randomly assigned to rate features of two animals (e.g., “dolphin” and “whale”) or two U.S. politicians (e.g., “George W. Bush” and “Hillary Clinton”). Participants were asked questions such as “Is a finch smart?” and responded by clicking either the “Yes” or “No” button on the screen. Each question was asked twice to measure response reliability. Thus, each participant saw 420 question trials.

### Experiment 2 Results

Clustering participants based on their feature ratings serves as a conceptual replication of Experiment 1. In the feature rating experiment, participant reliability was high with an average reliability of 86%. Similar to Experiment 1, subject responses likely reflect stable aspects of subjects’ conceptual representations. The number of concepts found was 6 to 16 for politicians 6 to 11 for animals, compared to 9 to 19 for politicians and 5 to 13 for animals in Experiment 1 (see Figure 10 in Supplementary Materials). Likewise, the ecological estimator results in 6 to 66 latent concepts in the population, compared to 6 to 50 in Experiment 1. Comparing the number of concepts for each word between experiments also results in high agreement, in both the MAP clustering (cosine similarity = 0.92) and ecological estimator (cosine similarity = 0.67). Such similar findings, despite a very different paradigm, provides convergent support for conceptual diversity.

**Figure 5.**
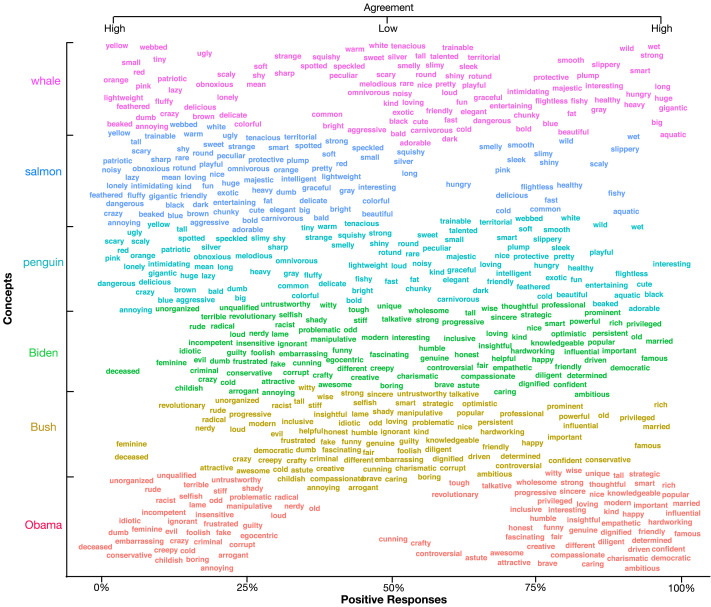
**A sampling of feature responses for 3 animals and 3 politicians (*y* axis).** The *x* axis plots the mean percentage of “yes” responses for a given feature. Features in the center show high disagreement among participants and are the primary features responsible for differing conceptual representations among participants.

Figure 5 shows agreement for a sample of features and concepts. Many features show near universal agreement among participants, but many also show large disagreement among participants. For example, most participants agreed that seals are not feathered but are slippery while disagreeing as to whether they are graceful. Likewise, most participants agreed that Trump is not humble and is rich, but there is high disagreement as to whether he is interesting. These sorts of disagreements likely reflect the different conceptual representations possessed by our participants, especially given the convergence between these findings and the similarity experiment. We note, however, that the results here could be due to differences between participants in the meaning of the features (e.g., what they think “interesting” refers to), though several theories of concepts (e.g., conceptual role theories, classical theories) have the meaning of “Trump” critically dependent on underlying features or related terms.

## DISCUSSION

We report statistical evidence of more than one variant of concepts in the population. In fact, we find that *most* people the average language user meets will not share their same concept. These results are unexpected in part because the measures we used are coarse. If one could gather an arbitrary amount of data, one might expect to find small differences between people: one interlocutor might have specific memories that make their representation idiosyncratic, perhaps different from anyone else. However, our experimental approach was based on judging similarities and features—not an exhaustive inventory of each person’s memories or associations—and we were nonetheless able to statistically justify measurably distinct representations, even for common nouns. If differences can be detected with these methods, it indicates that there is substantial variation in the population for lexical meanings. This variation exists despite the fact that people use the *same word* for each concept, and people are relatively unaware that others will tend to give differing similarity judgements.

However, our results do not support the notion that every single use of a concept is distinct or entirely idiosyncratic (Casasanto & Lupyan, 2015): subjects did group into clusters and did provide highly reliable responses across trials. We emphasize, though, that studies with more items, or items that focus more on corner cases, might find greater diversity than reported here. Future studies should examine the sensitivity of these results to target word and feature selection, with specific attention given to highly unrelated comparisons (e.g., is a train more similar to a dolphin or a slime mold). Moreover, the subject pool in our experiment was relatively homogeneous, and future studies of cultural differences may point to more diversity in word usage based on diversity of experience (Clark, 1998). Indeed, while our method allows us to quantify conceptual diversity, it does not pinpoint what specific representational differences drive this diversity. These differences may indeed go deep with respect to theories and interrelations between the concepts studied and others (Gelman & Legare, 2011; Medin & Rips, 2005; Murphy & Medin, 1985).

In general, theories of word learning and conceptual development will need to work out how human language users acquire distinct representations for shared words. In turn, theories of communication and language use (e.g., Grice, 1989; Wilson & Sperber, 2004) will need to address both differences in word referents, and lack of awareness of those differences. People’s general obliviousness to variation has important implications for productive discourse structure, and has been studied by psychologists in more general forms such as the false consensus effect (Marks & Miller, 1987) and egocentric bias (Ross & Sicoly, 1979). Fundamental misunderstandings may originate with individuals using the same word for distinct conceptual representations or under different contexts. Indeed, such differences in word meanings might underlie many classic philosophical questions (Piantadosi, 2015). Generally, our results may help to explain why “talking past each other” appears to be common in social and political debates: the common ground of even the most basic word meanings is only imperfectly shared.

## STATEMENT OF RELEVANCE

We demonstrate that conceptual variability is a common part of human conceptual systems, one that likely arises from experiential differences. Our results document substantial disagreement between people for word meanings, even for common concepts. These results suggest that fundamental conceptual differences in political and social discourse underlie many semantic disagreements.

## DATA AND MATERIALS AVAILABILITY

All data and code can be found at https://osf.io/bfwce/?viewonly=aaf1b62123ce4a31938e6a5b03e140cc.

## ACKNOWLEDGMENTS

The authors thank the Kidd Lab and the Computation and Language Lab for feedback. CK and SP received funding from DARPA (Machine Common Sense TA1, BAA number HR001119S0005) and NSF (Division of Research on Learning, Grant 2000759). CK received funding from Human Frontier Science Program (RGP0018/2016), Berkeley Center for New Media, The Jacobs Foundation, and Google Faculty Research Awards in Human-Computer Interaction.

## AUTHOR CONTRIBUTIONS

Louis Marti: Conceptualization; Formal analysis; Investigation; Methodology; Software; Visualization; Writing—Original draft; Writing—Review & editing. Shengyi Wu: Investigation; Visualization; Writing—Review & editing. Steven T. Piantadosi: Conceptualization; Funding acquisition; Methodology; Software; Supervision; Visualization; Writing—Review & editing. Celeste Kidd: Conceptualization; Funding acquisition; Methodology; Supervision; Writing—Review & editing.
