# Supplementary material for: Latent Diversity in Human Concepts
Source: Open Mind (Camb). 2023 Mar 9;7:79–92. doi: 10.1162/opmi_a_00072 (PMC10320827; doi:10.1162/opmi_a_00072)
Supplement: Supplementary file 1 [file opmi-07-79-s001.pdf]

SUPPLEMENTAL MATERIALS

Robustness to α

In order to assess the robustness of our general results to the value of α, we also ran our model using alpha values of half ($\alpha = .08$, reliability = 93%) and double ($\alpha = .32$, reliability = 80%) the value used in the main results. As Figure 8 and Figure 9 show, results are not substantially different, providing evidence that our main results are not overly sensitive to participant reliability. Specifically, our 87% observed reliability yielded $6 - 32$ concepts for animals and $11 - 50$ for political figures. Increasing reliability to 93% resulted in $6 - 80$ for animals and $24 - 127$ for politicians. Decreasing reliability to 80% resulted in $2 - 12$ for animals and $11 - 30$ for politicians.

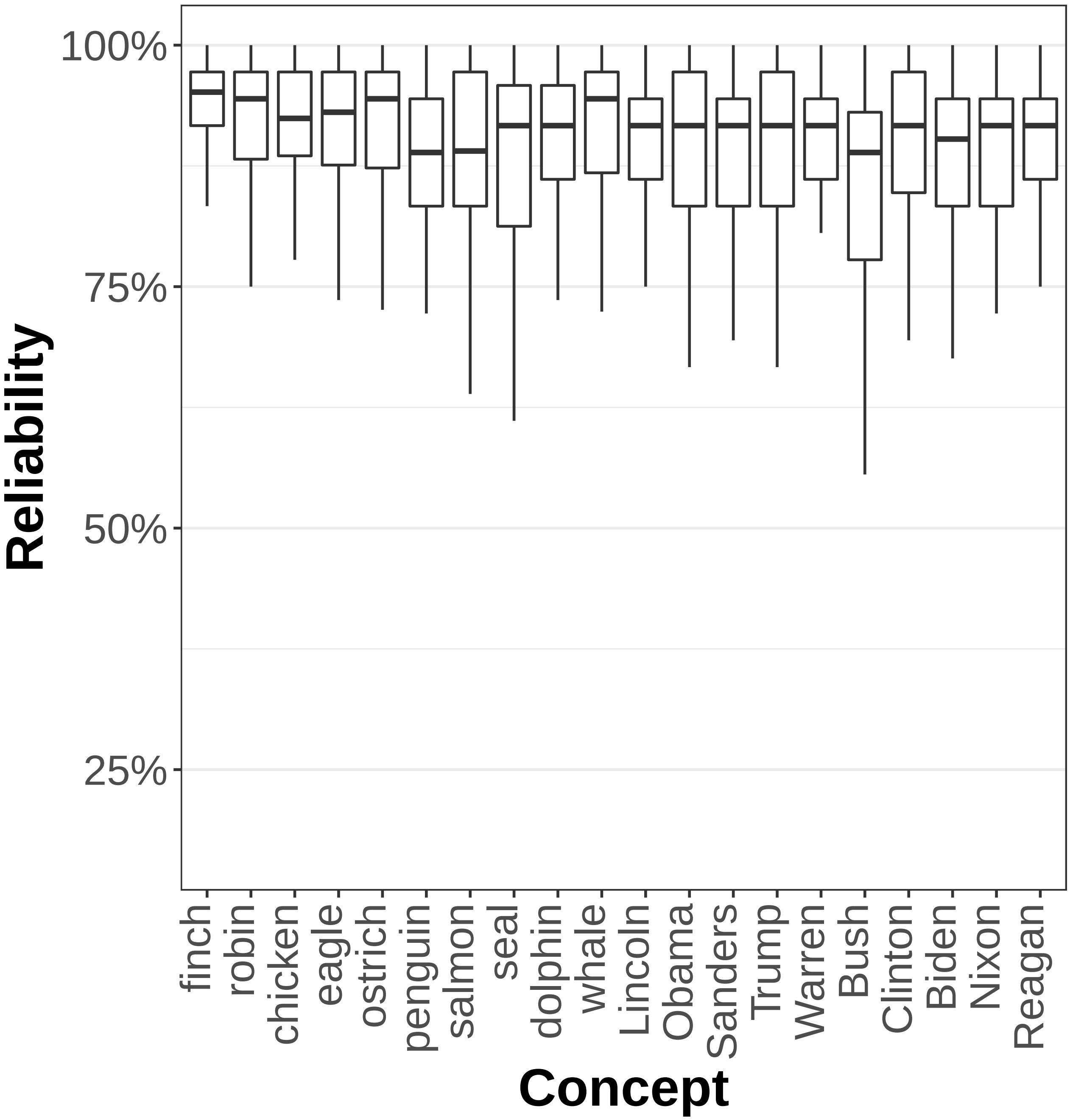

Figure 6. Participant reliability scores for each concept in Experiment 1. Boxes show the median 50% reliability quantiles. Median reliabilities for concepts range from 88.8% to 94.4%.

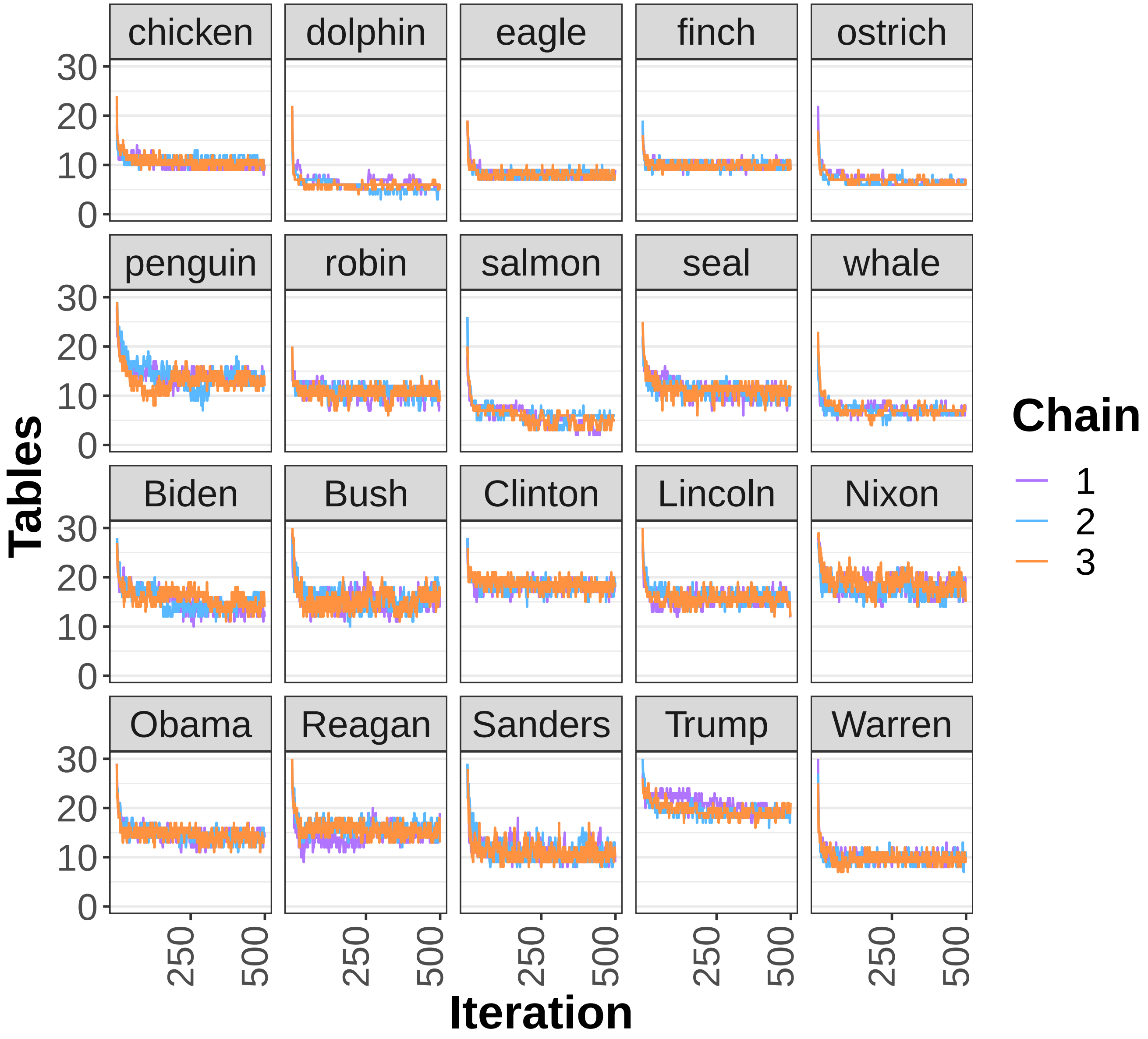

Figure 7. Convergence of the clustering model was assessed with multiple runs. Chains converge between 10 and 100 iterations.

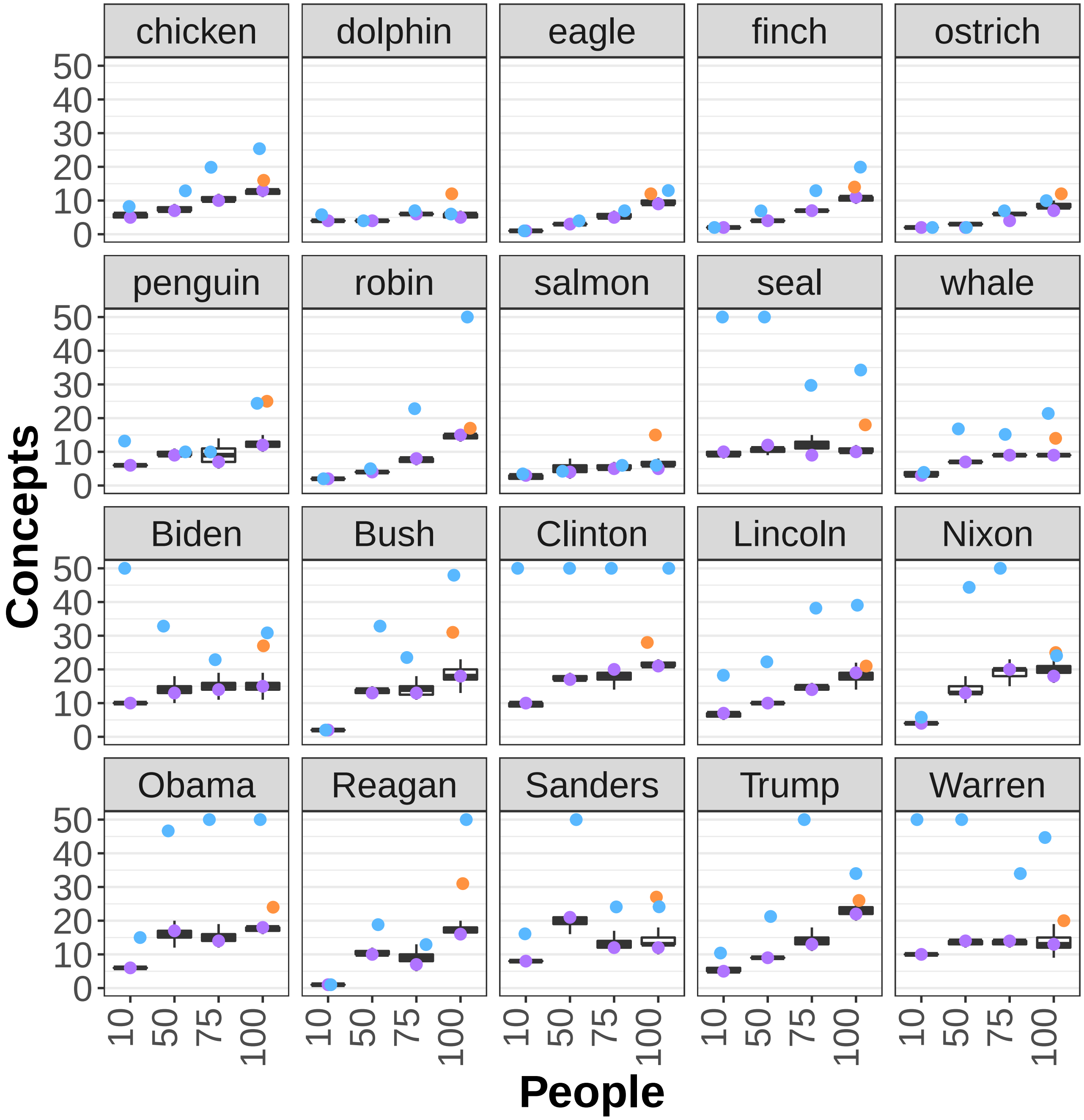

Figure 8. Estimated number of concepts (y-axis) depending on the number of people sampled (x-axis) for α = .08, reliability = 93%. Purple data points are the number of clusters for the *maximum a posteriori* clustering. Orange data points are the number of clusters for the MAP clustering with a uniform prior. Blue data points are a lower bound on the number of concepts estimated by the ecological estimator using the MAP clustering. The y-axis is set to the same range as Figure 4. Points higher than 50 are represented as 50.

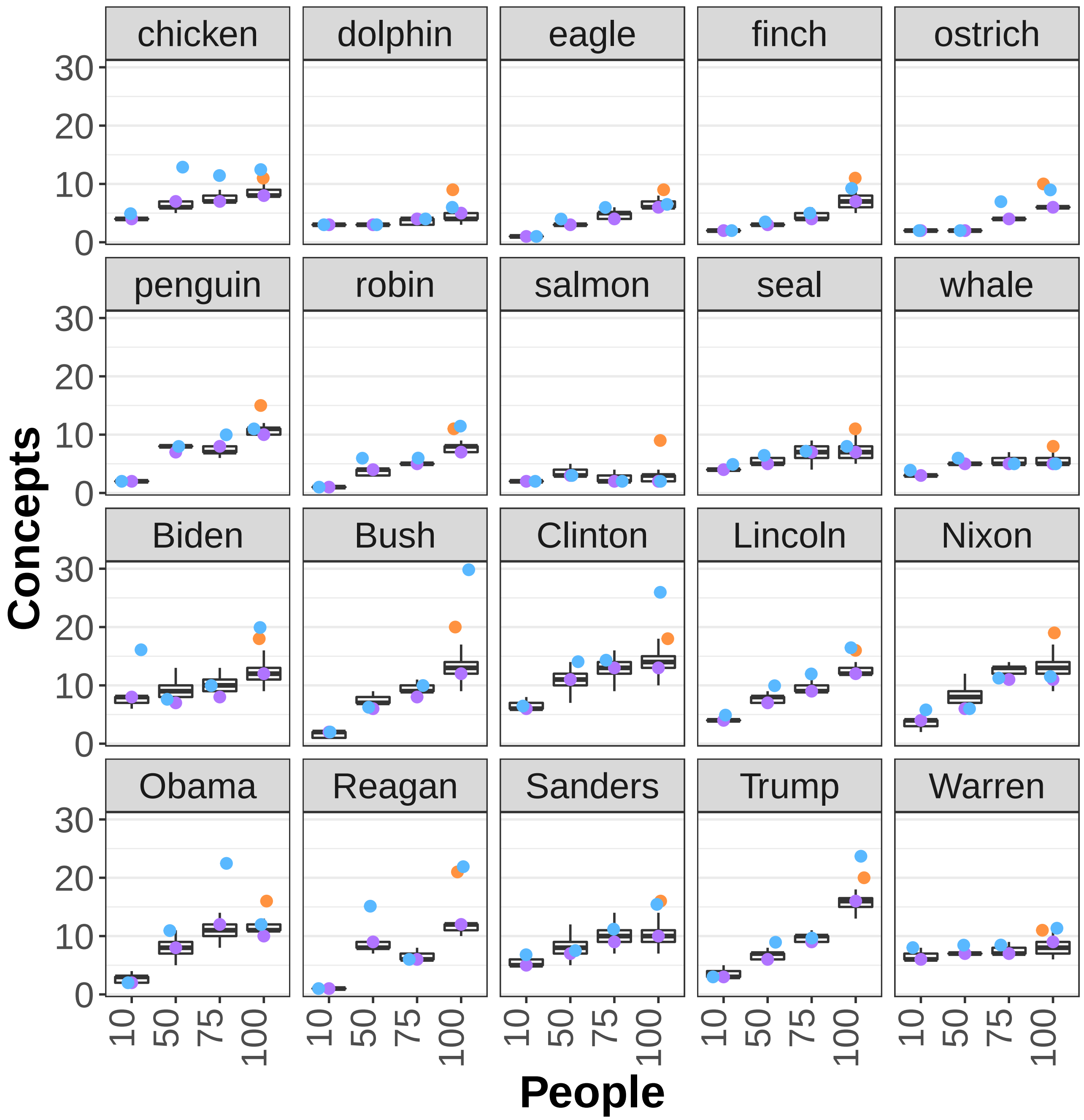

Figure 9. Estimated number of concepts (y-axis) depending on the number of people sampled (x-axis) for α = .32, reliability = 80%. Purple data points are the number of clusters for the *maximum a posteriori* clustering. Orange data points are the number of clusters for the MAP clustering with a uniform prior. Blue data points are a lower bound on the number of concepts estimated by the ecological estimator using the MAP clustering.

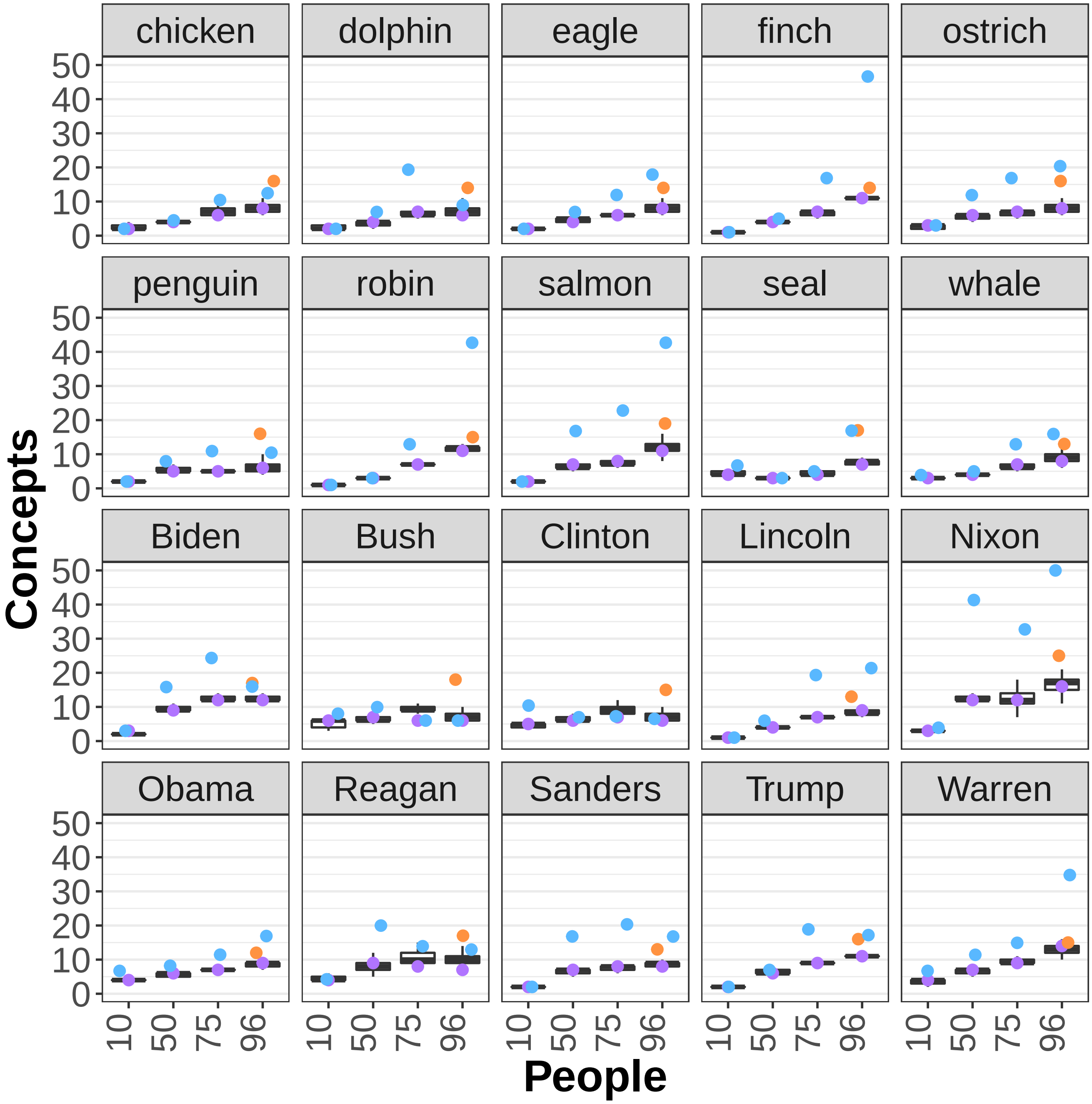

Figure 10. Experiment 2 - Estimated number of concepts (y-axis) depending on the number of people sampled (x-axis). Purple data points are the number of clusters for the *maximum a posteriori* clustering. Orange data points are the number of clusters for the MAP clustering with a uniform prior. Blue data points are a lower bound on the number of concepts estimated by the ecological estimator using the MAP clustering. The y-axis is set to the same range as Figure 4. Points higher than 50 are represented as 50.
